# The Meanings of Synesthetic Metaphors Closely Align With Crossmodal Correspondences

**DOI:** 10.1111/cogs.70209

**Published:** 2026-04-29

**Authors:** Mai Mori, Kimi Akita

**Affiliations:** ^1^ Department of English Linguistics Nagoya University

**Keywords:** Synesthetic metaphors, Crossmodal correspondences, Semantic differential technique, Conventionality, Emotion

## Abstract

Synesthetic metaphors are expressions in which the meaning of a word is transferred from one sense to another (e.g., *a bright sound*). They have often been discussed in relation to crossmodal correspondences, psychological phenomena where systematic associations are perceived between different senses (e.g., brightness and pitch). However, the relationship between these two phenomena has largely been treated as an assumption, with limited empirical examination. The present study investigated the extent to which synesthetic metaphors and crossmodal correspondences are related to each other. Specifically, through experiments using the semantic differential technique, it examined whether, for example, the sound represented by *a sweet sound* corresponds to the sound crossmodally associated with *a sweet taste*. It was found that the meanings of synesthetic metaphors were generally consistent with crossmodal correspondences; however, those of synesthetic metaphors that were acceptable or conventional (e.g., *a sour smell*) diverged from crossmodal correspondences (e.g., a smell associated with *a sour taste*). Furthermore, they were found to agree more closely with crossmodal correspondences on emotionally loaded scales, such as bad–good and dirty–clean, than on relatively emotion‐neutral scales, such as low‐pitched–high‐pitched. These findings indicate that the meanings of synesthetic metaphors, which generally align closely with crossmodal correspondences, may diverge from them when conventionalized and suggest that the two phenomena are emotionally mediated.

## 1 Introduction

### 1.1 Synesthetic metaphors

Synesthetic metaphors are traditionally defined as linguistic phenomena in which the meaning of a word is transferred from one sense (source sense) to another (target sense), as in *a bright sound* (Strik Lievers, 2015) and *This melody is smooth* (Winter & Strik‐Lievers, 2025).^1^ It has traditionally been argued that there is unidirectionality in how different sensory modalities are linguistically associated (Ullmann, 1959, 1962; Williams, 1976). Winter (2019) characterizes this tendency as “touch < taste < smell < sound/sight” (p. 101), where modalities on the left are more likely to serve as sources and those on the right as targets. Expressions that conform to this directionality (e.g., *a sweet melody*) are said to be not only more frequently used (Day, 1996; Ullmann, 1959) but also more natural and accessible than those that do not (e.g., *a melodious taste*) (Kusumi, 1988; Shen & Cohen, 1998; Werning, Fleischhauer, & Beşeiğlu, 2006). However, large‐scale meta‐analyses by Winter and Strik‐Lievers (2025) showed that relationships between sensory modalities should not be regarded as exceptionless unidirectionalities, but rather as asymmetries. In linguistics, synesthetic metaphors have long been vaguely assumed to be psychologically based, while the recent literature more specifically argues that they are analogous to crossmodal correspondences (Winter, 2019).

### 1.2 Crossmodal correspondences

Crossmodal correspondences are psychological phenomena defined as “a compatibility effect between attributes or dimensions of a stimulus (i.e., an object or event) in different sensory modalities (be they redundant or not)” (Spence, 2011, p. 973). They exist across all possible combinations of sensory modalities (Spence, 2011): for example, brightness and pitch (Marks, 1974); colors and touch (Ludwig & Simner, 2013); colors and tastes (Spence & Levitan, 2021); colors and odors (Gilbert, Martin, & Kemp, 1996); auditory frequency and haptic frequency (Yau, Olenczak, Dammann, & Bensmaia, 2009); pitch and tastes (Crisinel & Spence, 2010); pitch and odors (Speed, Croijmans, Dolscheid, & Majid, 2021); touch and tastes (Tu, Yang, & Ma, 2015); touch and odors (Speed et al., 2021); tastes and odors (Spence, 2022).

A wide variety of experimental methods have been employed to investigate crossmodal correspondences. According to Asano and Yokosawa (2020), these methods can be broadly divided into explicit and implicit approaches. Explicit approaches include direct matching tasks in which participants are asked to select the option that best matches the stimulus, and rating methods such as the semantic differential (SD) technique, in which impressions of stimuli are rated on multipoint scales constructed from pairs of antonymous adjectives (e.g., bad–good) (Osgood, Suci, & Tannenbaum, 1957). In contrast, implicit approaches involve tasks in which two stimuli are presented simultaneously, and participants are required to categorize one stimulus as quickly as possible while ignoring the other, as in the speeded classification task (Asano & Yokosawa, 2020, pp. 177–180).

Another important methodological difference lies in whether the stimuli are perceptual attributes or linguistic words. Martino and Marks (1999) conducted an experiment using the speeded classification task with both actual perceptual attributes (e.g., color patches of specific brightness) and linguistic stimuli (e.g., *BLACK*, *WHITE*) and uncovered that crossmodal correspondences occurred with both stimulus types. Specifically, the response time for judging whether a tone was high or low was shorter when participants were presented with congruent stimuli (e.g., a high‐pitched sound with *WHITE*) than when they were presented with incongruent stimuli (e.g., a high‐pitched sound with *BLACK*). Velasco, Woods, Marks, Cheok, and Spence (2016) exhibited a match between taste and shape using the SD method, and their taste stimuli were not perceptual stimuli but taste words. Lee and Spence (2022) pointed out that linguistic stimuli (e.g., *sweet, bitter*) had often been employed in studies on crossmodal correspondences, particularly those involving taste, because there are practical challenges in using perceptual stimuli, such as tastant solutions. They argue that the results obtained with linguistic and perceptual stimuli are comparable, suggesting that, at least in the domain of taste, both types of stimuli may induce crossmodal correspondences.

### 1.3 Crossmodal correspondences as a motivation for synesthetic metaphors

In linguistics, crossmodal correspondences have been discussed as a psychological basis motivating synesthetic metaphors. O'Malley (1957) highlighted the fact that both crossmodal correspondences (“intersense analogy” in his terms) and synesthetic metaphors are widely shared among individuals. Winter (2019) echoed this argument, noting that both involve environmentally coupled senses such as gustation and olfaction, which usually co‐occur in daily life, and are not necessarily perceived consciously. The latter feature contrasts with synesthesia, which is perceived vividly, automatically, and consciously. Winter further argues that synesthetic metaphors should instead be called “crossmodal metaphors,” as, for example, expressions such as *a bright sound* and *a dark sound* may reflect the crossmodal correspondence between auditory pitch and visual luminance.

This view has also been taken by some psychologists. Di Stefano, Murari, and Spence (2022) stated that “language has been used in a literary context in order to convey crossmodal associations between scent and sound,” clearly indicating that they regard synesthetic metaphors as linguistic manifestations of crossmodal correspondences (p. 159). Asano and Yokosawa (2020) also support the view that crossmodal correspondences underlie the comprehension of synesthetic metaphors, albeit in part.

Furthermore, Marks (1982a, b) does not merely assume that crossmodal correspondences motivate synesthetic metaphors but, to the best of our knowledge, provided the only empirical examinations of this claim, as also noted by Winter (2019). Marks (1982a) elucidated, using rating experiments, that poetic synesthetic metaphors linking light and sound (e.g., *bright thunder*) systematically mapped brightness onto loudness or pitch and, conversely, mapped loudness or pitch onto brightness. Specifically, *bright thunder* was rated as louder or higher‐pitched than *dim thunder*, while *loud glow* was rated as brighter than *soft glow*. Marks (1982b) likewise investigated poetic synesthetic metaphors linking light and sound (e.g., *the murmur of the gray twilight*) and found a reliable correlation between brightness and loudness, by employing an experimental paradigm involving the manipulation of actual light and sound stimuli rather than subjective ratings. Marks (1982a) stated that the similarities observed across sensory modalities constitute a part of human implicit knowledge, which is expressed through language.

### 1.4 Crossmodal correspondences as linguistically motivated phenomena

Synesthetic metaphors and crossmodal correspondences have several properties in common, and it seems reasonable to assume a certain interrelation between them. However, the causal relationship between the two phenomena is not self‐evident. In fact, several studies in psychology propose an opposite causal relationship, arguing that synesthetic metaphors may motivate crossmodal correspondences.

Imschloss and Kuehnl (2019) maintain that crossmodal correspondences between music and touch arise because adjectives typically used for tactile sensations (e.g., *soft*, *smooth*, *hard*, *rough*) can also be applied to music (e.g., *a soft melody, a rough sound*), thereby giving rise to crossmodal associations at the semantic level. Martino and Marks (1999) termed this the “semantic‐coding hypothesis.” This idea constitutes one of the traditional classifications of crossmodal correspondences, together with “structural correspondences,” in which correspondences are mediated by shared neural substrates such as loudness and brightness, and “statistical correspondences,” in which correspondences are formed through the repeated coexperience of different senses (e.g., small size and high‐pitched sounds) in our daily lives (Spence, 2011).

Indeed, some studies experimentally showed that synesthetic metaphors can influence crossmodal correspondences. Dolscheid, Shayan, Majid, and Casasanto (2013) conducted a nonlinguistic experiment investigating interference between pitch and the height or thickness of visual lines among Dutch speakers, who describe pitch as *high*/*low*, and Farsi speakers, who describe it as *thin*/*thick*. They found that, for Dutch speakers, height influenced pitch, whereas for Farsi speakers, thickness had an effect. Dolscheid, Çelik, Erkan, Küntay, and Majid (2020) further compared Dutch speakers with Turkish speakers, who, like Farsi speakers, describe pitch as *thin*/*thick*, and revealed that space–pitch associations differed between these groups.

### 1.5 Lack of empirical research demonstrating the correlation between synesthetic metaphors and crossmodal correspondences

As discussed in Sections 1.3 and 1.4, there are two opposing perspectives on the relationship between synesthetic metaphors and crossmodal correspondences: crossmodal correspondences motivating synesthetic metaphors versus synesthetic metaphors motivating crossmodal correspondences. The former view has been taken in both linguistics and psychology, while the latter view has also been present in psychology. The first issue with these previous studies is that both views essentially remain theoretical assumptions. Dolscheid et al. (2013, 2020) are exceptional in that they actually provided experimental evidence that speakers of different languages differed in their nonlinguistic associative patterns, thereby going beyond theoretical assumptions and addressing the causal relationship from synesthetic metaphors to crossmodal correspondences.

The second, and most crucial, issue is that causality has been addressed prematurely, without establishing a correlation between synesthetic metaphors and crossmodal correspondences. Although Marks (1982a, b) examined the correlation between the two phenomena, the scope of his investigations were confined to highly poetic synesthetic metaphors (e.g., *sound of coming darkness*) and to the crossmodal correspondences between brightness and loudness or pitch. Therefore, the findings cannot be generalized to the broader relationship between synesthetic metaphors and crossmodal correspondences. Consequently, the observations on the relationship between synesthetic metaphors and crossmodal correspondences remain primarily grounded in superficial similarities, such as the fact that both involve environmentally coupled senses, are widely shared, and are not necessarily consciously perceived (O'Malley, 1957; Winter, 2019). Thus, the present study broadens the scope to nonpoetic synesthetic metaphors and a diverse range of crossmodal correspondences and aims to demonstrate a systematic correlation between synesthetic metaphors and crossmodal correspondences, rather than jumping straight to questions of causality.

## 2 Methods

The present study investigates whether sources and targets of synesthetic metaphors are consistent with crossmodal correspondences. For example, in order to demonstrate that *a bright sound* is based on the crossmodal correspondence between brightness and high‐pitched sounds, it must be shown that *a bright sound* refers to a high‐pitched sound (Marks, 1982a). However, previous research has tended to discuss the targets of synesthetic metaphors at the sense level, such as audition, and relatively little attention has been paid to more fine‐grained targets (Kusumi, 1988; Winter, 2019).

**Fig. 1:**
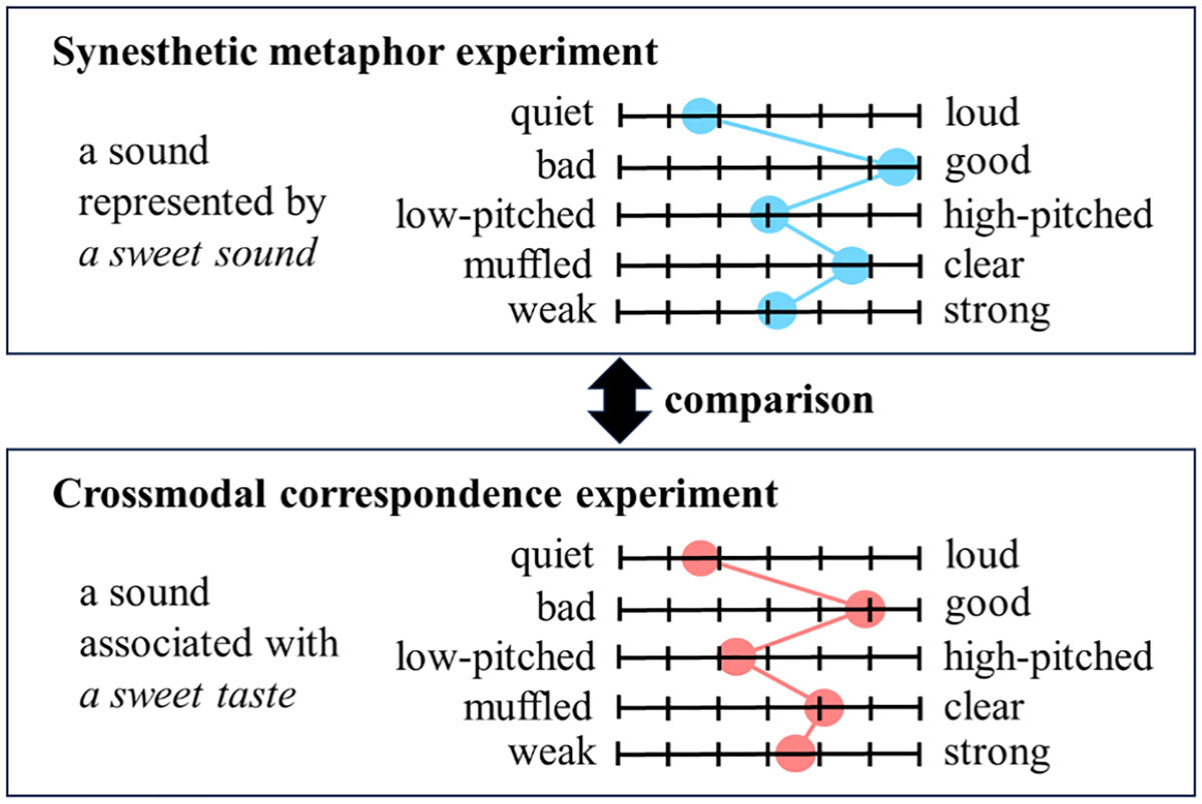
An example of the experimental procedure.

To address this gap, we quantified the targets of various synesthetic metaphors (synesthetic metaphor experiment) and the relevant crossmodal correspondences (crossmodal correspondence experiment) using the same set of SD scales. The ratings for synesthetic metaphors and crossmodal correspondences were compared to assess whether consistency really exists between the two phenomena. An example of the experimental procedure is shown in Fig. 1.

The stimuli for the synesthetic metaphor experiment took the [adjective + noun] forms. Crossmodal correspondences were examined indirectly by presenting participants with linguistic stimuli, such as *a sweet taste*, asking them to imagine the sound, color, touch, taste, and smell associated with their referent, and then having them rate those imagined features.

### 2.1 Participants

#### 2.1.1 Participants in the synesthetic metaphor experiment

To minimize participants’ burden, the entire set of stimuli for the synesthetic metaphor experiment was divided into three separate subsets, and the three subsets were answered by 30 distinct participants recruited for each (*N* = 90 in total; 49 female, 40 male, 1 nonbinary; *M*_age_ = 48.40, range 20–72). Each participant rated 100 items (20 stimuli × 5 scales). Only English monolinguals residing in the United States were recruited via Prolific. Participants received £2.50 as compensation for their participation.

#### 2.1.2 Participants in the crossmodal correspondence experiment

The stimuli for the crossmodal correspondence experiment were also divided into three. A total of 30 distinct participants who were English monolinguals residing in the United States were recruited for each (*N* = 90 in total; 57 female, 32 male, 1 undisclosed; *M*_age_ = 45.11, range 24–73) via Prolific. The compensation was £2.50. No participant participated in the synesthetic metaphor experiment. It would be ideal that synesthetic metaphors and corresponding crossmodal correspondences were rated by the same participants; however, because the designs of the two experiments were highly similar, they were kept separate to prevent influence from each other.

### 2.2 Stimuli

#### 2.2.1 Stimuli for the synesthetic metaphor experiment

In constructing the synesthetic metaphor stimuli, adjectives associated with each sense were selected under consistent criteria and then systematically combined with sensory nouns. This method was adopted to ensure that the number of stimuli did not vary across adjectives’ senses. Similar procedures have also been employed in some previous experimental studies (Kusumi, 1988; Winter & Strik Lievers, 2023). For the selection of adjectives, a dataset was constructed by integrating the Lancaster Sensorimotor Norms (Lynott, Connell, Brysbeart, Brand, & Carney, 2020) with the SUBTLEX‐US frequency list (Brysbaert & New, 2009, Brysbaert, New, & Keuleers, 2012).

The Lancaster Sensorimotor Norms provide ratings indicating the extent to which concepts (i.e., words) are experienced through each sense (audition, gustation, touch, olfaction, vision). For example, the five‐sense perceptual norms for the adjective *bright* are as follows: audition = 0.18, gustation = 0.00, touch = 0.35, olfaction = 0.06, and vision = 4.71. The dataset also contains “modality exclusivity,” which quantifies the extent to which a concept is experienced through a single modality, expressed as a proportion between 0 (completely multimodal) and 1 (completely unimodal). Since *bright* is predominantly associated with the visual modality compared to the others, its five‐sense modality exclusivity score is high (0.89). In contrast, the word *harsh* has more balanced ratings across modalities (auditory = 3.39, gustatory = 1.28, haptic = 1.39, olfactory = 1.00, visual = 3.44), resulting in a relatively low modality exclusivity score of 0.23. The SUBTLEX‐US frequency list is a large‐scale lexical database based on movie and television subtitles, which provides frequency information for over 70,000 English words and includes part‐of‐speech information.

From the integrated dataset, adjectives were selected that exhibited high modality exclusivity (i.e., a strong association with a single sensory modality) and ranked within the top 25% in log10 frequency among adjectives belonging to each modality.^2^ These criteria were established to exclude multimodal adjectives (e.g., *intense*) and select adjectives that belong exclusively to a single modality and are frequently used. The validity of the selection was subsequently examined through manual inspection. Items that did not clearly represent a specific sensory attribute (e.g., *colored* for vision) or were semantically similar to another adjective with higher modality exclusivity (e.g., *creaking* [lower in modality exclusivity] vs. *squeaky* [higher in modality exclusivity]) were excluded, and another adjective meeting the criteria was selected as a replacement. The selected adjectives are shown in Table 1.

**Table 1:** The adjectives selected for stimuli

Visual	Auditory	Haptic	Gustatory	Olfactory
*pink*	*squeaky*	*sticky*	*salty*	*stinking*
*white*	*muffled*	*fluffy*	*sweet*	*fragrant*
*purple*	*noisy*	*soft*	*sour*	*perfumed*

Each of these adjectives was crossmodally combined with four of the following sensory nouns taken from Kusumi (1988): *color* (vision), *sound* (audition), *touch* (touch), *taste* (gustation), and *smell* (olfaction). Although abstract nouns such as *sight* or *look* could have been used for vision, this study followed previous research (Kusumi, 1988; Winter & Strik Lievers, 2023) in adopting *color*. This choice is further supported by the fact that colors have been extensively investigated in research on crossmodal correspondences (Ludwig & Simner, 2013; Spence, 2020; Spence & Levitan, 2021; Hidaka & Shimoda, 2014). The final set of synesthetic metaphor stimuli is presented in Table 2.

**Table 2:** Stimuli for the synesthetic metaphor experiment

_Adjective_^Noun^	*Color*	*Sound*	*Touch*	*Taste*	*Smell*
*Pink*		*a pink sound*	*a pink touch*	*a pink taste*	*a pink smell*
*White*		*a white sound*	*a white touch*	*a white taste*	*a white smell*
*Purple*		*a purple sound*	*a purple touch*	*a purple taste*	*a purple smell*
*Squeaky*	*a squeaky color*		*a squeaky touch*	*a squeaky taste*	*a squeaky smell*
*Muffled*	*a muffled color*		*a muffled touch*	*a muffled taste*	*a muffled smell*
*Noisy*	*a noisy color*		*a noisy touch*	*a noisy taste*	*a noisy smell*
*Sticky*	*a sticky color*	*a sticky sound*		*a sticky taste*	*a sticky smell*
*Fluffy*	*a fluffy color*	*a fluffy sound*		*a fluffy taste*	*a fluffy smell*
*Soft*	*a soft color*	*a soft sound*		*a soft taste*	*a soft smell*
*Salty*	*a salty color*	*a salty sound*	*a salty touch*		*a salty smell*
*Sweet*	*a sweet color*	*a sweet sound*	*a sweet touch*		*a sweet smell*
*Sour*	*a sour color*	*a sour sound*	*a sour touch*		*a sour smell*
*Stinking*	*a stinking color*	*a stinking sound*	*a stinking touch*	*a stinking taste*	
*Fragrant*	*a fragrant color*	*a fragrant sound*	*a fragrant touch*	*a fragrant taste*	
*Perfumed*	*a perfumed color*	*a perfumed sound*	*a perfumed touch*	*a perfumed taste*	

#### 2.2.2 Stimuli for the crossmodal correspondence experiment

The crossmodal correspondence experiment used the same set of adjectives as in the synesthetic metaphor experiment (Table 1). Stimuli were created by pairing each adjective with the same‐sense noun from the synesthetic metaphor experiment (e.g., *a squeaky sound*). The visual adjectives (i.e., *pink*, *purple*, and *white*) were presented with the visual noun *color* in parentheses (e.g., *pink [color]*), as they can function as nouns and phrases, such as *a pink color*, might sound redundant.

### 2.3 SD scales

The same set of SD scales was used in the synesthetic metaphor and crossmodal correspondence experiments. Five scales were employed for each of the five senses, the bad–good scale being the only scale shared among all senses. The selection of these scales was based on previous studies on each sense. Only scales used in at least three studies were selected.^3^ The SD scales are shown in Table 3.

**Table 3:** The SD scales used

Visual	Auditory	Haptic	Gustatory	Olfactory
dark–bright	quiet–loud	dry–moist	sweet–not sweet	dirty–clean
red–not red	low‐pitched–high‐pitched	rough–smooth	bitter–not bitter	masculine–feminine
blue–not blue	muffled–clear	hard–soft	sour–not sour	mild–harsh
green–not green	weak–strong	angular–round	salty–not salty	stale–fresh
bad–good	bad–good	bad–good	bad–good	bad–good

### 2.4 Instructions

In the synesthetic metaphor experiment, they were presented with the constructed synesthetic metaphors (e.g., *a soft color*) and asked the question, “What kind of *x* does this expression represent?” Here, *x* was replaced with the noun corresponding to the relevant sense (i.e., *color*, *sound*, *touch*, *taste*, or *smell*). Participants were asked to rate the attributes each synesthetic metaphor represents on 7‐point SD scales, with 1 and 7 representing the opposing adjectives in Table 3. They were instructed to respond intuitively, even if the expression felt unfamiliar or unnatural to them.

In the crossmodal correspondence experiment, the question format was “What kind of *x* matches *y*?,” where *x* was replaced with one of the nouns (e.g., *color*), and *y* was replaced by a constructed unimodal phrase (e.g., *a sweet taste*). Participants were asked to rate each stimulus on the above 7‐point SD scales.

### 2.5 Procedures

**Fig. 2:**
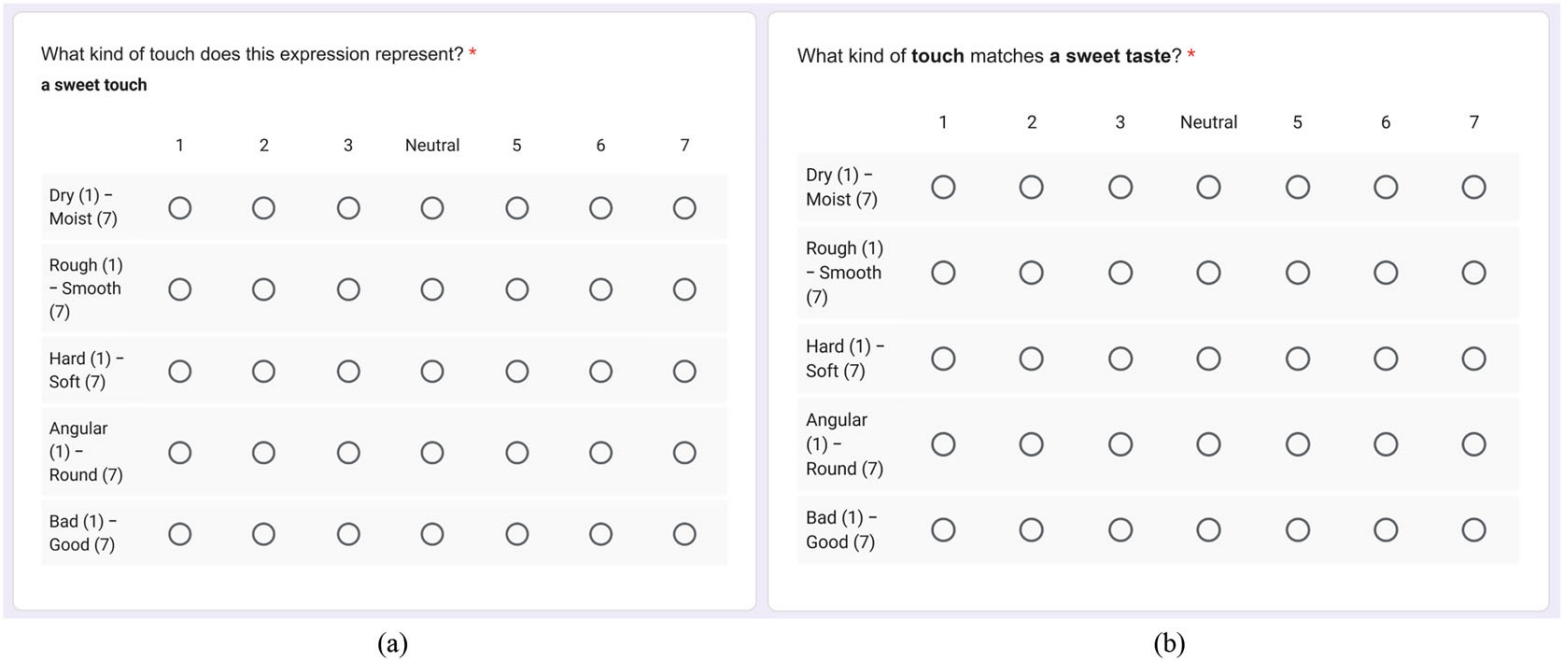
Example questions (a) Synesthetic metaphor experiment (b) Crossmodal correspondence experiment.

All experiments were created using Google Forms, as shown in Fig. 2. Prior to participation, participants were informed about the nature of the experiments, and only those who provided consent proceeded to the test pages. The synesthetic metaphor experiment was conducted from September 23 to 24, 2025, and the crossmodal correspondence experiment on September 24, 2025.

**Fig. 3:**
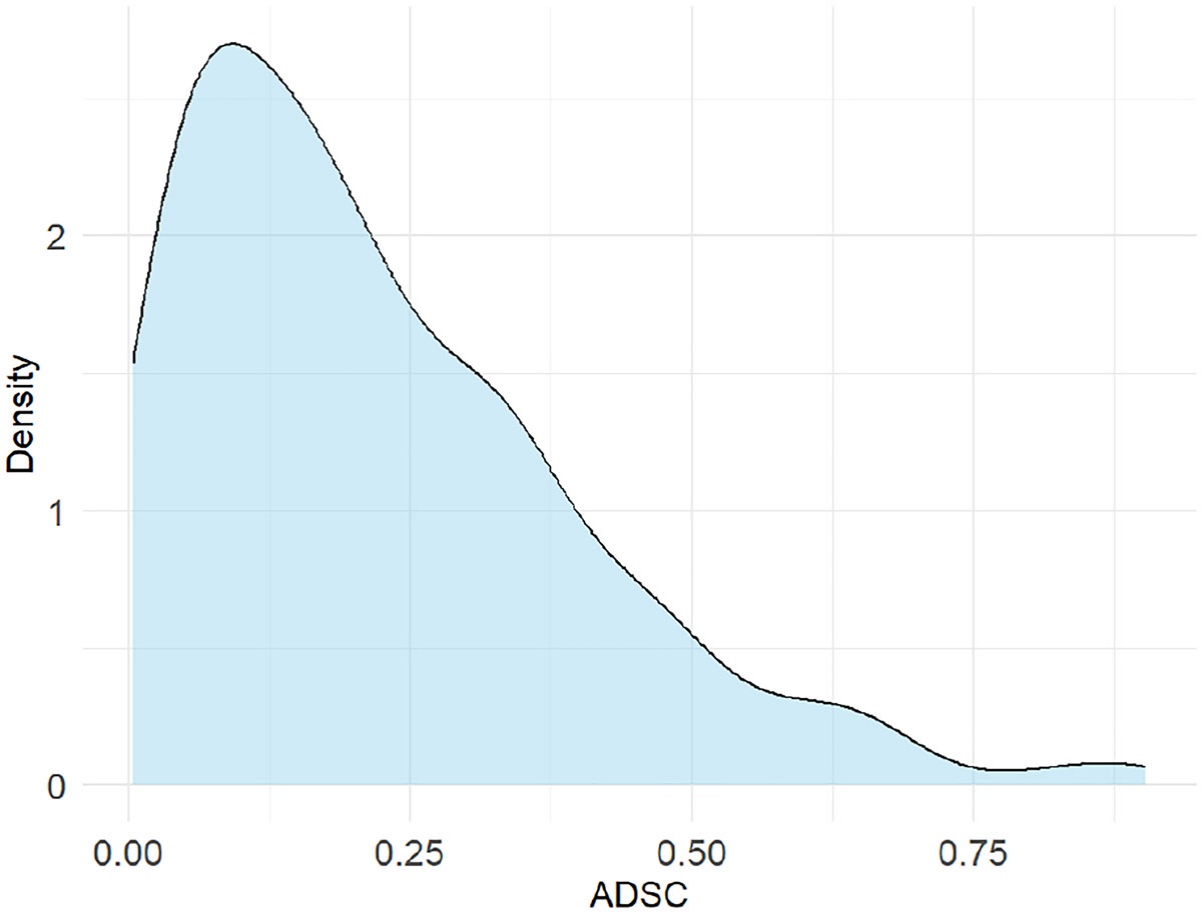
Distribution of ADSCs across all scales.

### 2.6 Data cleaning

To screen out participants who did not respond with sufficient attention, an attention‐check question asking “Are you currently focused on the experiment?” was included twice in each experiment. The order of the response options (“yes” and “no”) was reversed each time. Participants who selected “no” on either question were not included in the dataset. Responses from seven out of 90 participants were excluded from the synesthetic metaphor experiment, and those from one participant were excluded from the crossmodal correspondence experiment. No participants were excluded because of straightlining responses.

### 2.7 Statistical analysis

All statistical analyses were conducted using R version 4.4.0 (R Core Team, 2024). All plots were generated using the ggplot2 package version 3.5.1 (Wickham, 2016). Linear mixed effects models were fitted using the lme4 package version 1.1.37 (Bates, Maechler, Bolker, & Walker, 2015) and the lmerTest package version 3.1.3 (Kuznetsova, Brockhoff, & Christensen, 2017). Generalized Variance Inflation Factor values were calculated using the car package version 3.1.3 (Fox & Weisberg, 2019). All stimuli, instructions, experimental forms, data, and R scripts are available on this study's Open Science Framework (OSF) repository: https://osf.io/fbqs4/.

## 3 Results

### 3.1 Descriptive statistics

The differences in ratings between synesthetic metaphors and crossmodal correspondences on each scale—calculated as the absolute differences of mean *z*‐scored ratings for synesthetic metaphors minus mean *z*‐scored ratings for crossmodal correspondences (hereafter, ADSCs)—were 0.22 on average (*SD* = 0.18). For example, the mean rating for *a fluffy sound* on the quiet (1)–loud (7) scale was 2.17 (mean *z*‐scored rating = −0.88), indicating a relatively quiet sound. The mean rating on the same scale for the sound crossmodally associated with *a fluffy touch* was 2.00 (mean *z*‐scored rating = −0.83), which also indicated a relatively quiet sound, and the absolute difference between the two mean *z*‐scored ratings resulted in 0.05 (|(−0.88) − (−0.83)|). The distribution of ADSCs across all scales is shown in Fig. 3.

The pairs of synesthetic metaphors and crossmodal correspondences showing the smallest and largest ADSCs are presented in Table 4. The overall distributions of raw ratings for synesthetic metaphors and crossmodal correspondences are shown in Appendices A1, A2, A3, A4, A5. The means of the raw ratings and standard deviations for all stimuli on all scales are provided on this study's OSF repository.

**Table 4:** Synesthetic metaphors and crossmodal correspondences which have the top and bottom five ADSCs

Smallest ADSC			
	SM and CC	Scale	ADSC
1	*a purple taste*	sweet–not sweet	0.0038
	a taste associated with *purple (color)*		
2	*a soft smell*	dirty–clean	0.0058
	a smell associated with *a soft touch*		
3	*a noisy taste*	salty–not salty	0.0060
	a taste associated with *a noisy sound*		
4	*a perfumed color*	blue–not blue	0.0080
	a color associated with *a perfumed smell*		
5	*a purple sound*	muffled–clear	0.0082
	a sound associated with *purple (color)*		

Largest ADSC			
	SM and CC	Scale	ADSC
1	*a stinking touch*	dry–moist	0.9024
	a touch associated with *a stinking smell*		
2	*a sour smell*	dirty–clean	0.8709
	a smell associated with *a sour taste*		
3	*a white sound*	muffled–clear	0.8571
	a sound associated with *white (color)*		
4	*a sour smell*	stale–fresh	0.7921
	a smell associated with *a sour taste*		
5	*a sour sound*	bad–good	0.6783
	a sound associated with *a sour taste*		

*Note*. Synesthetic metaphors are referred to as SM, and crossmodal correspondences as CC.

Several representative associations reported in the literature on crossmodal correspondences were replicated in the present data. For instance, *a* *noisy* *sound* was associated with a relatively bright color, which Marks (1974) showed using pure tones and color paper varying in brightness from black through white. The results also exhibited that *a sweet taste* was associated with a relatively rounded touch, which was reported in Velasco et al.’s (2016) study using the SD method. The results that *pink (color)* was rated as matching a relatively sweet taste replicated the associative relationship between pink and sweetness (Spence & Levitan, 2021; Wan et al., 2014).

### 3.2 Relationship between synesthetic metaphor ratings and crossmodal correspondence ratings

**Fig. 4:**
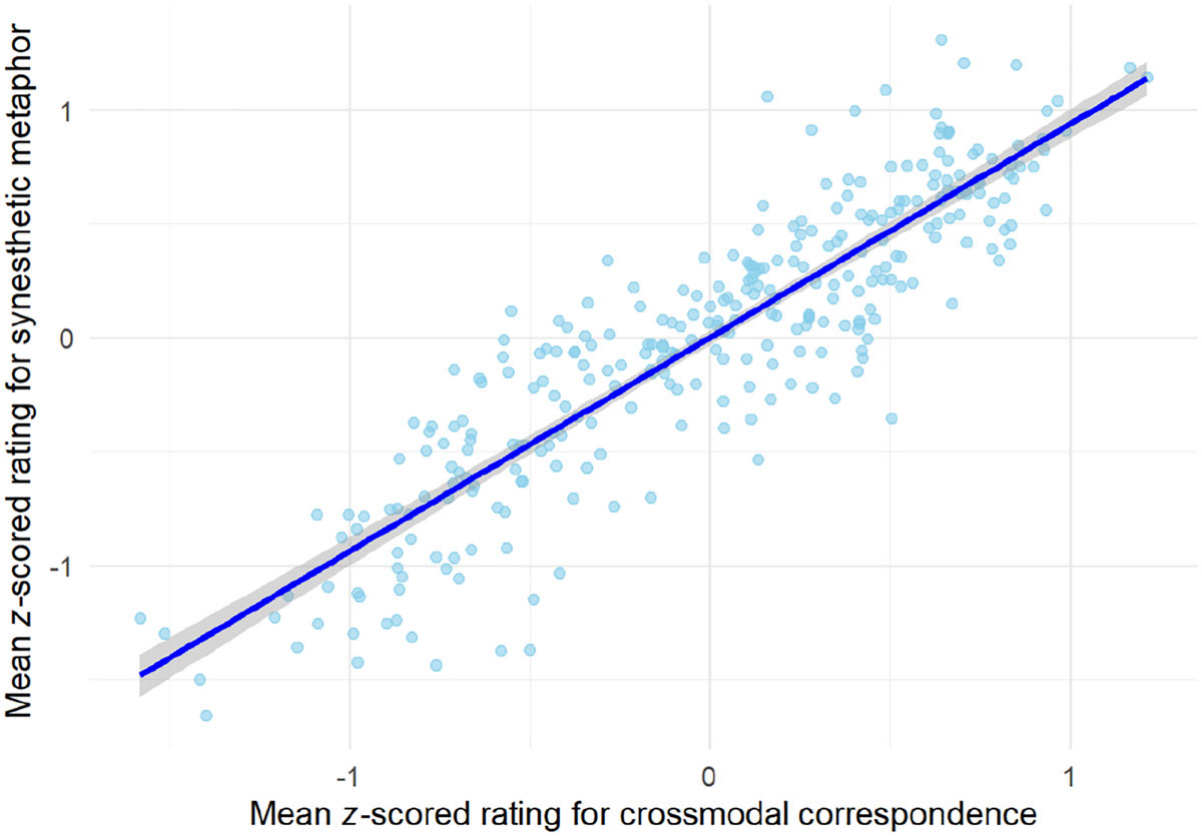
Mean *z*‐scored ratings for synesthetic metaphors and crossmodal correspondences.

The present section statistically examines the overall degree of agreement between the ratings for synesthetic metaphors and crossmodal correspondences. Fig. 4 shows mean *z*‐scored ratings for synesthetic metaphors and crossmodal correspondences on each scale.

The Pearson correlation coefficient between the mean *z*‐scored ratings for synesthetic metaphors and those for crossmodal correspondences was *r*(298) = .89 (*p* < .001), indicating a strong positive correlation between the two phenomena.

### 3.3 Acceptability

The results presented in Section 3.2 indicated that the ratings for synesthetic metaphors and crossmodal correspondences showed strong agreement overall. However, as shown in Table 4, some pairs of synesthetic metaphors and crossmodal correspondences exhibited relatively large ADSCs. As introduced in Section 1.1, the acceptability of synesthetic metaphors differs depending on which sensory modalities are combined and how (e.g., *a sweet melody* vs. *a melodious taste*). We examined whether ADSC varied as a function of the acceptability of synesthetic metaphors.

Two competing predictions can be made regarding the relationship between acceptability and ADSC. One possibility is a negative correlation in which more acceptable synesthetic metaphors are more strongly motivated by crossmodal correspondences and, therefore, show smaller ADSCs. The other possibility is a positive correlation in which more acceptable synesthetic metaphors, through becoming conventionalized, acquire more idiosyncratic meanings and consequently show larger ADSCs. For further discussion of conventionality, see Section 3.4.

A total of 30 native speakers of English residing in the United States who did not participate in the main experiments (18 female, 12 male; *M*_age_ = 42.33, range 20–73) were recruited via Prolific and rated the acceptability of the synesthetic metaphors on a 7‐point scale ranging from 1 (not acceptable at all) to 7 (perfectly acceptable), after giving informed consent. They were paid £1.25. The data are available on OSF.

**Fig. 5:**
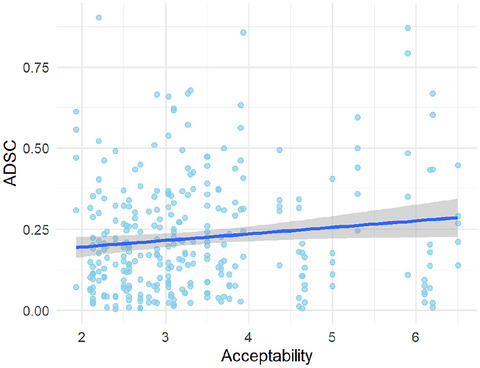
Mean acceptability ratings and ADSCs.

The mean acceptability across all the synesthetic metaphors was 3.36 (*SD* = 2.26). Fig. 5 shows the relationship between the mean acceptability rating of each synesthetic metaphor and ADSC on each scale.

The Pearson correlation coefficient between the mean acceptability ratings and ADSCs was *r*(298) = .13 (*p* = .03), indicating a weak but significant positive correlation. This shows that synesthetic metaphors with higher acceptability (e.g., *a soft sound*) deviate more from crossmodal correspondences.

### 3.4 Conventionality

We also examined the conventionality of synesthetic metaphors as a possible predictor of ADSC. As with acceptability, there are two possible predictions about the relationship between conventionality and ADSC. One prediction is that more conventionalized synesthetic metaphors are more strongly grounded in perception and, therefore, exhibit smaller ADSCs, as anonymous reviewers pointed out. The other prediction is that highly conventional synesthetic metaphors have developed idiosyncratic meanings and, therefore, show larger ADSCs. Strik Lievers (2016) noted that in German and related languages, the synesthetic‐metaphorical use of *süß* “sweet,” as in *süßesten Tönen* “sweetest tone,” is so conventionalized that it is no longer perceived as synesthetic or metaphorical, but rather as literal and evaluative (i.e., “pleasant”). Indeed, the *Oxford English Dictionary* gives “pleasant” as a separate sense of the word *sweet* (Winter & Strik‐Lievers, 2025). These observations allow us to infer that the more conventionalized synesthetic metaphors are, the more idiosyncratic they tend to be, and consequently, the more likely they are to diverge from their psychological basis.

The conventionality of the synesthetic metaphors was measured using both ratings and attestation frequency in the *Corpus of Contemporary American English* (COCA) (Davies, 2008).^4^ For the ratings, 30 native speakers of English residing in the United States who did not participate in the main experiments or the acceptability ratings (15 female, 15 male; *M*_age_ = 42.73, range 19–74) were recruited via Prolific. After providing informed consent, participants rated the synesthetic metaphors on a 7‐point scale ranging from 1 (not conventional) to 7 (perfectly conventional).

**Fig. 6:**
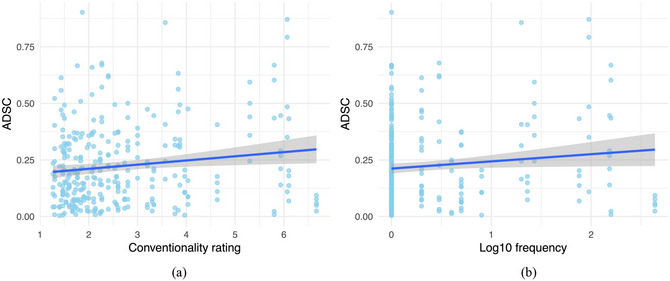
(a) Mean conventionality ratings and ADSCs (b) Log10 frequency in COCA and ADSCs.

The mean conventionality across all the synesthetic metaphors was 2.66 (*SD* = 2.08), and the mean raw attestation frequency in COCA was 17.28 (*SD* = 63.41), including all their inflected forms (e.g., *sweet sound*, *sweeter sound*, *sweetest sound*, *sweet sounds*, *sweeter sounds*, *sweetest sounds*). Fig. 6 shows the relationship between the mean conventionality ratings and ADSCs, and  that between log10 frequency and ADSCs.

Both conventionality ratings (*r*(298) = .14, *p* = .01) and log10 frequency (*r*(298) = .12, *p* = .04) showed significant positive correlations with ADSCs, which suggest that the more conventional a synesthetic metaphor is, the more it tends to diverge from the relevant crossmodal correspondence.

### 3.5 SD scales

**Fig. 7:**
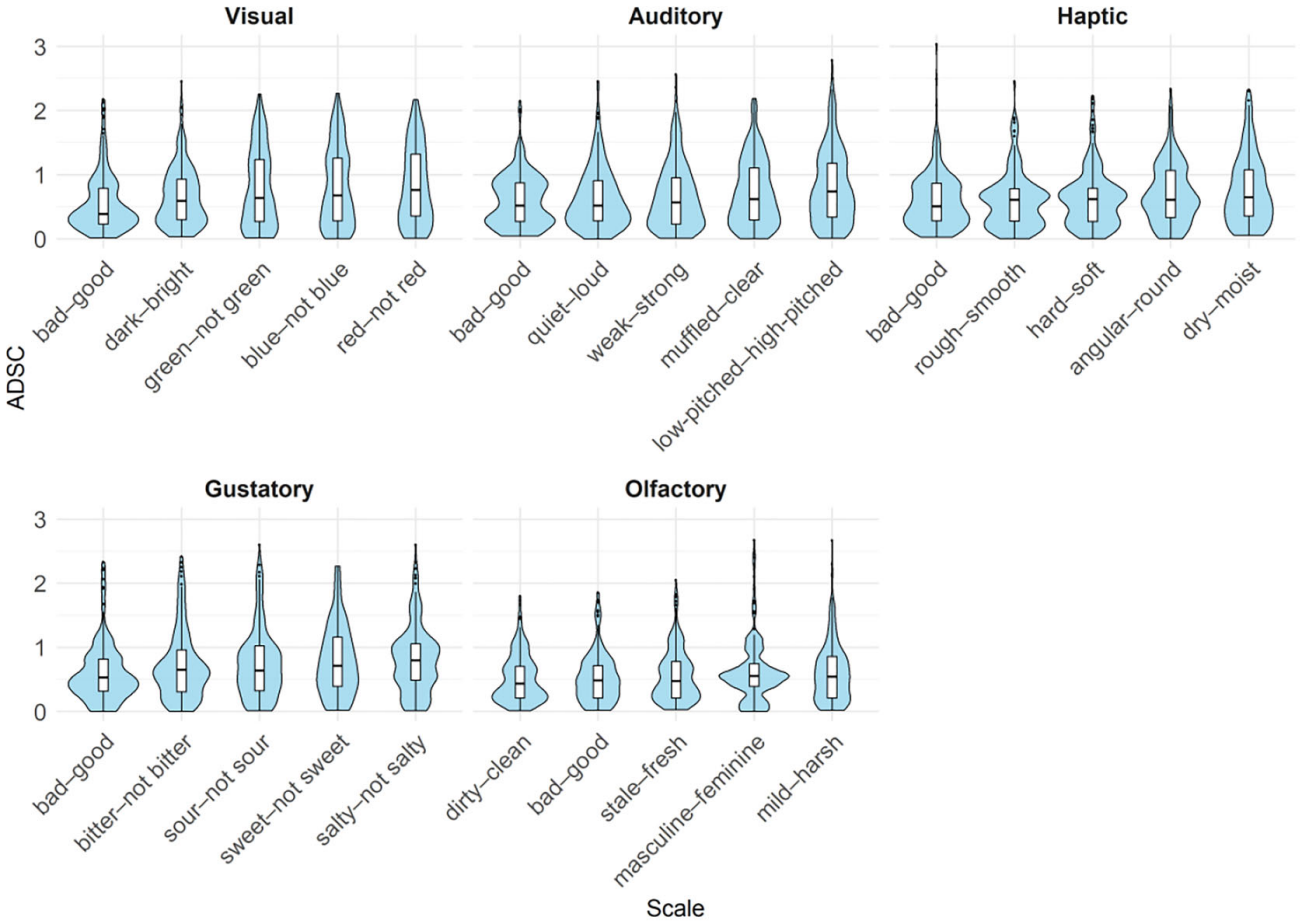
ADSCs across SD scales.

An additional exploratory analysis was conducted to examine the potential influence of the SD scales on ADSC. Fig. 7 shows ADSCs on each SD scale, separately for each target sense. Here, ADSCs were calculated as each participant's ratings of synesthetic metaphors minus the mean ratings for crossmodal correspondences, in anticipation of the subsequent statistical modeling.The SD scales are arranged from left to right within each target sense in ascending order of ADSCs.

For example, ADSCs tended to be smaller on the bad–good scale than on the red–not red scale for *a sweet color*. To examine these scale‐wise differences, a linear mixed effects model was fitted for each sense. The dependent variable was ADSC, and the SD scale and conventionality rating were included as fixed effects. Random effects consisted of stimulus and participant, with a random slope of conventionality rating by participant, as in (1).

(1) $$\mathrm{ADSC}∼\mathrm{SDScale}+\mathrm{Conventionality}+\left(1|\mathrm{Stimulus}\right)+\left(1+\mathrm{Conventionality}|\mathrm{Participant}\right)$$

The random slope in the auditory model was removed because it caused a singular fit issue, and, as including both acceptability and conventionality produced GVIF values above 3.5, only conventionality was entered into the model. In each model, the reference level was set to the bad–good scale, which generally exhibited the smallest ADSCs. Tables 5, 6, 7, 8–9 show the differences between the bad–good scale and the other SD scales.

**Table 5:** Regression outputs for the visual SD scales, with the bad–good scale as the reference level

Scale	*b*	*SE*	*t*	*p*
dark–bright	0.10	0.04	2.66	< .01^**^
green–not green	0.23	0.04	6.20	< .001^***^
blue–not blue	0.26	0.04	7.02	< .001^***^
red–not red	0.28	0.04	7.33	< .001^***^

*** *p* < .001; ** *p* < .01.

**Table 6:** Regression outputs for the auditory SD scales, with the bad–good scale as the reference level

Scale	*b*	*SE*	*t*	*p*
quiet–loud	0.03	0.03	0.94	.35
weak–strong	0.07	0.03	1.94	.05
muffled–clear	0.13	0.03	3.62	< .001***
low‐pitched–high‐pitched	0.21	0.03	6.05	< .001***

*** *p* < .001.

**Table 7:** Regression outputs for the haptic SD scales, with the bad–good scale as the reference level

Scale	*b*	*SE*	*t*	*p*
rough–smooth	0.01	0.03	0.33	.74
hard–soft	0.04	0.03	1.23	.22
angular–round	0.12	0.03	3.62	< .001***
dry–moist	0.18	0.03	5.60	< .001***

*** *p* < .001.

**Table 8:** Regression outputs for the gustatory SD scales, with the bad–good scale as the reference level

Scale	*b*	*SE*	*t*	*p*
bitter–not bitter	0.12	0.04	3.16	< .01**
sour–not sour	0.14	0.04	3.90	< .001***
salty–not salty	0.21	0.04	5.68	< .001***
sweet–not sweet	0.20	0.04	5.49	< .001***

*** *p* < .001; ** *p* < .01.

**Table 9:** Regression outputs for the olfactory SD scales, with the bad–good scale as the reference level

Scale	*b*	*SE*	*t*	*p*
dirty–clean	−0.01	0.03	−0.49	.63
stale–fresh	0.03	0.03	1.17	.24
masculine–feminine	0.07	0.03	2.47	.02*
mild–harsh	0.08	0.03	2.67	.01*

*p < .05.

Within each sense, ADSCs on some SD scales were comparable to those observed for the bad–good scale. Specifically, the quiet–loud and weak–strong scales for audition, the rough–smooth and hard–soft scales for touch, and the dirty–clean and stale–fresh scales for olfaction exhibited ADSCs that were as small as those on the bad–good scale. The other SD scales (e.g., dry–moist) exhibited larger ADSCs than the bad–good scale.

## 4 Discussion

The current experiments made three major findings. First, there was an overall agreement between synesthetic metaphors and crossmodal correspondences with respect to rating patterns (Section 3.2). Second, the meanings of relatively acceptable or conventional synesthetic metaphors diverged from crossmodal correspondences (Sections 3.3 and 3.4). Third, the degree of agreement between synesthetic metaphors and crossmodal correspondences varied across SD scales (Section 3.5). The possible implications of these findings are discussed in detail below.

### 4.1 The meanings of synesthetic metaphors are consistent with crossmodal correspondences

Previous studies have discussed the superficial similarities between synesthetic metaphors and crossmodal correspondences, focusing on their prevalence, environmentally coupled modalities, and the lack of necessarily conscious perception (O'Malley, 1957; Winter, 2019). On the other hand, the present results suggest deeper correspondences between the two phenomena. Specifically, it turned out that the sources and targets of synesthetic metaphors were consistent with relevant crossmodal correspondences. For example, *a soft sound* represented a sound that was relatively good, low‐pitched, muffled, quiet, and weak, and the sound associated with *a soft touch* was likewise relatively good, low‐pitched, muffled, quiet, and weak. These findings confirm the previously implicit assumption that synesthetic metaphors are closely linked to crossmodal correspondences.

### 4.2 Synesthetic metaphors drift away from crossmodal correspondences through conventionalization

The acceptability and conventionality ratings obtained in the present study showed a very strong correlation (*r*(58) = .96, *p* < .001). This suggests that acceptable synesthetic metaphors tend to be used frequently and, as a result, become conventionalized. Through conventionalization, synesthetic metaphors may gradually develop idiosyncratic meanings, which may be abstract or closely associated with specific contexts of use. For instance, *a sour smell*, which was rated relatively acceptable (*M* = 5.90, *SD* = 1.69) and conventional (*M* = 6.07, *SD* = 1.70), had a relatively large absolute difference from the smell associated with *a sour taste* on the dirty–clean and stale–fresh scales, as shown in Table 4. *A sour smell* was judged as representing a much dirtier and staler smell than the smell associated with *a sour taste*. This instance suggests that *a sour smell* has become associated with a specific context of rotten food and has acquired the idiosyncratic meaning of dirtiness. In contrast, *a sour taste* may evoke citrus fruits, such as lemons or limes, whose associated smells are not strongly linked to dirtiness. As this example suggests, by acquiring idiosyncratic meanings as linguistic expressions, synesthetic metaphors may drift away from crossmodal correspondences.

At the same time, it should be noted that, although the acceptability ratings, conventionality ratings, and log10 frequency in COCA each showed significant positive correlations with ADSCs, the correlation coefficients were relatively small. Given that the present study used a small set of experimental stimuli, stronger effects of acceptability and conventionality may emerge with a larger and more diverse set of stimuli.

### 4.3 Synesthetic metaphors and crossmodal correspondences may share the same emotional processes

The SD scales also affected the relationship between synesthetic metaphors and crossmodal correspondences. For example, *a sweet touch* showed a closer match to the touch associated with *a sweet taste* on the bad–good, hard–soft, and rough–smooth scales than on the angular–round and dry–moist scales. Among all the SD scales, the weak–strong, quiet–loud, rough–smooth, hard–soft, dirty–clean, and stale–fresh scales exhibited as small ADSCs as the bad–good scale.

A shared property of these scales is that their polar adjectives have contrasting valence values. Valence refers to the positive or negative meaning associated with a word or concept and has been quantified through rating studies (Warriner, Kuperman, & Brysbaert, 2013). For instance, *rough* has a mean valence rating of 3.68 on a scale from 1 (very negative) to 9 (very positive), indicating that it is relatively negative, whereas *smooth* has a mean valence rating of 6.42, indicating that it is relatively positive. The valence contrast between *rough* and *smooth* is, therefore, 2.74. The mean valence contrast was 3.50 (*SD* = 0.77) for *bad* and *good* and the adjective pairs constituting scales on which ADSCs were as small as those on the bad–good scale. In contrast, the mean valence contrast of the other scales (e.g., low‐pitched–high‐pitched) was 1.57 (*SD* = 0.65).^5^ In other words, the relationship between synesthetic metaphors and crossmodal correspondences was stronger on emotionally loaded SD scales that mapped onto a positive–negative dimension than on relatively emotion‐neutral SD scales, such as low‐pitched–high‐pitched.

Linear mixed effects models were employed to predict ADSCs from these valence contrasts represented by the scales, with the random effects of stimulus and participant. It was revealed that sharper valence contrasts significantly predicted smaller ADSCs (visual model: *b* = −0.03, *SE* = 0.01, *t* = −3.12, *p* < .01; auditory model: *b* = −0.04, *SE* = 0.01, *t* = −3.49, *p* < .001; haptic model: *b* = −0.05, *SE* = 0.01, *t* = −5.68, *p* < .001; olfactory model: *b* = −0.03, *SE* = 0.01, *t* = −3.54, *p* < .001).^6^ In addition, the correlation between the mean *z*‐scored ratings for synesthetic metaphors and crossmodal correspondences was stronger when only the emotionally loaded scales were included (*r*(130) = .93, *p* < .001) than when all scales were considered (*r* = .89, Section 3.2).

Emotion has been highlighted as a key factor in both synesthetic metaphors and crossmodal correspondences. With regard to synesthetic metaphors, Winter (2019) argues that *a sweet melody* is used primarily because *sweet* carries positive valence. In this view, synesthetic metaphors do not necessarily involve metaphorical mappings; rather, adjectives are sufficiently abstracted into evaluative meanings to modify nouns from different sensory modalities. Similarly, Kusumi (1988) proposes that synesthetic metaphors are grounded in the affective (pleasant–unpleasant) meanings of adjectives. In research on crossmodal correspondences, emotion has been emphasized as one of the mediating factors (Di Stefano & Spence, 2024). For example, the association between music and color (Palmer, Schloss, Xu, & Prado‐León, 2013), music and taste (Guetta & Loui, 2017; Park & Kim, 2024), and music and smell (Levitan, Charney, Schloss, & Palmer, 2015) have been attributed to the emotion they share. As additional support for these previous studies, the results of the present study revealed that synesthetic metaphors and crossmodal correspondences were more closely related on emotionally loaded scales. These results suggest that the two phenomena may share a common emotional basis.

## 5 Conclusion

This study has demonstrated the systematic correlation between synesthetic metaphors and crossmodal correspondences, which has not been given sufficient attention in previous research. Specifically, it was found that the sources and targets of synesthetic metaphors are consistent with the patterns of crossmodal correspondences. However, it was also found that the more acceptable and conventional a synesthetic metaphor is, the weaker its alignment with crossmodal correspondences tends to be. The particularly strong linkage observed on emotionally loaded scales suggests that the two phenomena may be supported by their shared emotional grounds.

As a future direction, the current findings should be tested with a larger stimulus set. In the present study, three adjectives per sensory modality were combined with a single modality‐specific noun. Expanding the range of both adjectives and nouns would be beneficial for a more comprehensive investigation. It is also important to replicate our findings using nonlinguistic paradigms because linguistic stimuli, such as those used in the present study, may capture only semantic‐level associations, leaving open the possibility that more perceptually grounded crossmodal correspondences may differ. For instance, it would be worthwhile to compare brain activity during the comprehension or production of synesthetic metaphors with that observed when crossmodal correspondences are elicited.

It remains necessary to examine whether there is a causal relationship beyond a mere consistency between synesthetic metaphors and crossmodal correspondences. Although the strong agreement between the two phenomena observed for the emotionally loaded scales was interpreted by assuming the emotional grounds shared by the two phenomena, the possibility that one may motivate or influence the other cannot be ruled out (Dolscheid et al., 2013, 2020). It is even possible that the relationship varies across individual synesthetic metaphors and crossmodal correspondences: some may be linked through the emotional mediation, whereas others may involve a more direct influence of synesthetic metaphors on nonlinguistic crossmodal correspondences, or vice versa. In other words, these three possibilities may coexist.

In addition, since the present study's focus was limited to crossmodal correspondences, future research should also investigate the relationship between synesthetic metaphors and other phenomena involving multisensory interactions, such as synesthesia (Kusumi, Yokosawa, Asano, & Harashima, 2023) or multisensory integration (Ronga, Bazzanella, Rossi, & Iannetti, 2012). In particular, synesthesia is a phenomenon whose relationship with crossmodal correspondences has been actively discussed (Martino & Marks, 2001). Thus, examining the relationship between synesthetic metaphors and synesthesia would be another promising direction for future research.

## Funding

This study was partly supported by the Grant‐in‐Aid for Scientific Research (B) (no. 23H00627) and Nagoya University Graduate School of Humanities Grant for Research Activities.

## Conflicts of interest

The authors declare that they have no conflicts of interest.

## Ethics approval statement

The experimental data were collected strictly adhering to APA's ethical principles, and ethical approval was granted by the Ethics Committee of Nagoya University Graduate School of Humanities (NUHM‐25‐14).

## Figures and Tables

**Fig. A1:**
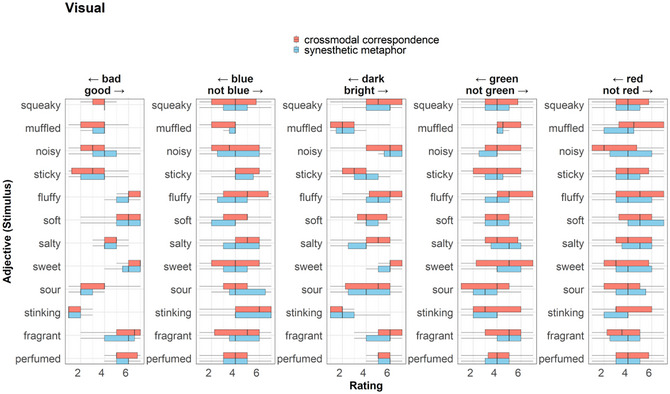
Distributions of raw ratings for synesthetic metaphors and crossmodal correspondences on the visual scales, grouped by adjectives (e.g., ratings for the crossmodal correspondence for a sweet taste [red] and the synesthetic metaphor a sweet color [blue] are given under the adjective sweet).

**Fig. A2:**
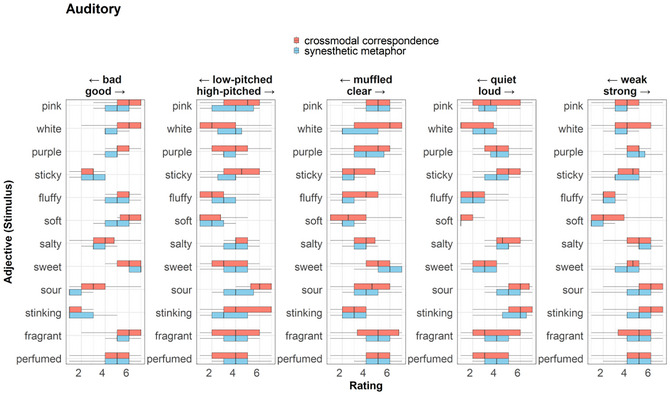
Distributions of raw ratings on the auditory scales.

**Fig. A3:**
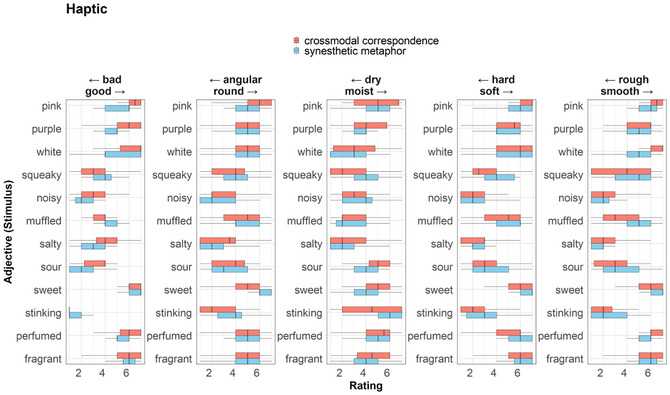
Distributions of raw ratings on the haptic scales.

**Fig. A4:**
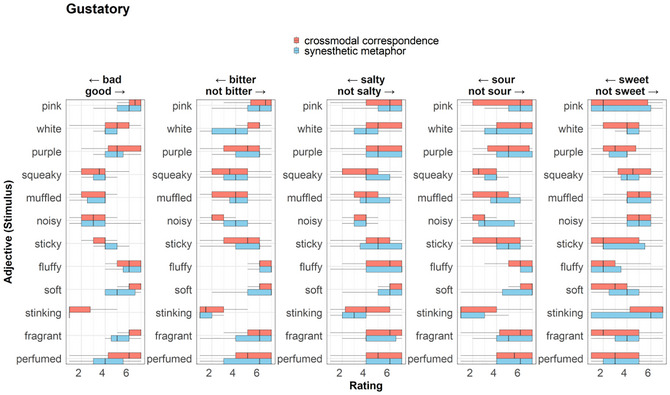
Distributions of raw ratings on the gustatory scales.

**Fig. A5:**
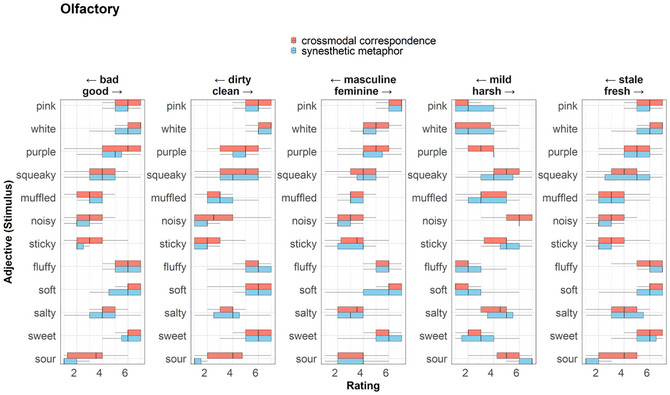
Distributions of raw ratings on the olfactory scales.

## Data Availability

All stimuli, data, and R scripts are available on the study's OSF repository (https://osf.io/fbqs4/).

## Notes

1 Werning, Fleischhauer, and Beşeiğlu (2006) distinguish “weak” and “strong synesthetic metaphors.” The former consists of a perceptual adjective and an amodal noun (e.g., *cold heart*), whereas the latter consists of a perceptual adjective and a perceptual noun (e.g., *cold smell*). Synesthetic metaphors discussed in this paper are the latter, strong ones.

2 Adjectives were selected in descending order of modality exclusivity within each sense. As a result, the ranges of modality exclusivity for the selected adjectives were as follows: visual: 0.93–0.94, auditory: 0.76–0.83, haptic: 0.54–0.59, gustatory: 0.52–0.59, olfactory: 0.80–0.89.

3 The references used for the selection of SD scales are as follows: visual: Oyama, Tanaka, and Haga (1963), Shimizu, Doizaki, and Sakamoto (2014), Shinomori and Takata (2025); auditory: Hülsmeier, Schell‐Majoor, Rennies, and van de Par (2014), Kankubo and Kobayashi (2007), Ueda (1988); haptic: Doizaki, Iiba, Okatani, and Sakamoto (2015), Shimizu et al. (2014), Yoshida (1968); gustatory: Schiffman and Erickson (1971), Turoman, Velasco, Chen, Huang, and Spence (2018), Velasco, Woods, Marks, Cheok, and Spence (2016); olfactory: Cunningham and Crady (1971), Dalton, Maute, Hikichi, and Izumi (2008), Wakata and Saito (2014). Many of these studies have examined actual perceptual, rather than linguistic, stimuli from each sensory modality using the SD method.

4 We owe this rating‐based analysis of conventionality to an anonymous reviewer.

5 Valence contrast could not be computed for some SD scales because the relevant adjectives were either absent from Warriner, Kuperman, and Brysbaert (2013) or appeared in the form of [adjective–not adjective] (e.g., red–not red). The mean valence contrast was calculated using six out of the seven emotionally loaded scales and five out of the 14 relatively emotion‐neutral scales for which valid valence data were available.

6 Because the gustatory scales, except for the bad–good scale, take the form of [adjective–not adjective], it was not possible to model them in terms of valence contrast.
